# The effect of tactile versus auditory stimulation on reducing pain associated with invasive procedures among preterm neonates at NICUs

**DOI:** 10.1186/s12887-025-05979-w

**Published:** 2025-08-16

**Authors:** Entsar Shehata Mohammed, Seham Mohammed Elmwafie, Fatma Sayed Abdelaziz Mohamed, Safaa Salah Ismail

**Affiliations:** 1 Beni-Suef University, Beni-Suef City, Egypt; 2 Pediatric Nursing Department, Faculty of Nursing, Beni-Suef University, Beni-Suef City, Egypt; 3 Helwan University, Helwan City, Egypt

**Keywords:** Auditory stimulation, Invasive procedures, Tactile stimulation, Pain, Preterm neonate

## Abstract

**Background:**

Preterm neonates often undergo invasive procedures during the period spent in neonatal intensive care units (NICUs), yet their ability to deal with pain is limited due to their immature nervous systems. Managing pain efficiently in this age is crucial, as recurrent or improperly controlled pain can have over-time influences on their neurological and physiological development. The study aimed to determine the effect of tactile versus auditory stimulation on reducing pain associated with invasive procedures among preterm neonates at NICUs. The study hypothesized that preterm neonates who receive tactile stimulation will exhibit lower pain scores than those who receive auditory stimulation.

**Methods:**

A comparative (three-group) quasi-experimental design included 90 preterm neonates divided equally into three groups (tactile stimulation group = 30, auditory stimulation group = 30, and control group = 30) at NICU affiliated with Beni-Suef University Hospital. Neonatal Infant Pain Scale (NIPS) was utilized four times: before, during, immediately after, and after five minutes of each invasive procedure to collect data between March and August 2024.

**Results:**

During the invasive procedure, preterm neonates in the tactile stimulation group showed the lowest mean scores across pain indicators, including facial expression (0.30 ± 0.466), breathing pattern (0.37 ± 0.490), arm movement (0.53 ± 0.507), leg movement (0.43 ± 0.504), state of arousal (0.70 ± 0.450), cry (0.63 ± 0.615), oxygen saturation (0.37 ± 0.490), and heart rate (0.60 ± 0.498). The total NIPS mean score was highest in the control group (8.47 ± 1.196), indicating severe pain, followed by the auditory stimulation group (5.57 ± 1.431), indicating moderate pain, and lowest in the tactile stimulation group (3.50 ± 1.225), indicating mild pain. A statistically significant difference in NIPS scores was observed among the three groups during and after the procedure (*p* < 0.05), while no significant difference was found before the procedure (*p* > 0.05).

**Conclusion:**

Both tactile and auditory stimulation can significantly reduce pain scores in preterm neonates undergoing painful invasive procedures and are effective in improving physiological stability and neurobehavioral outcomes of premature neonates.

**Implications to practice:**

NICUs should involve tactile and auditory stimulation interventions in the routine nursing care for hospitalized preterm neonates as safe and effective non-pharmacological techniques of pain management. So, replicating the study in other geographical settings is essential, to generalize findings to different settings.

## Introduction

Preterm birth or prematurity can be defined as birth that occurs before the gestational age of 37 weeks [1, 2]. The underdeveloped state of preterm infants isn’t only a result of their atypical early-life environment, but it is additionally related to medical issues involving pain exposure [3]. The International Association for the Study of Pain defines pain as ‘’an unpleasant emotional and sensory experience relevant to either existing or potential tissue damage’’ [4]. Preterm neonates routinely encounter an average of 12–17 invasive procedures daily. A small percentage encountered more than 3000 painful procedures during their NICU stay, and as a result, pain exposure is higher [5, 6]. In addition to short-term physiological instability and pain sensitivity, prolonged negative effects are strongly associated with repetitive pain-related stress in premature infants [7, 8]. Preterm neonates have the right to effective, safe, and efficient pain relief to enhance their health outcomes [9–11]. Non-pharmacological treatment refers to approaches that do not involve the use of pharmaceuticals such as behavioral and environmental ones [12]. Auditory and tactile stimulations are the most widely used sensory measures to reduce preterm neonates’ pain and stress [13]. In fact, the premature Infant at NICUs receives a great deal of touch when fed, carried, bathed, or held. Such forms of stimulation provide needed excitement to the central nervous system (CNS), and when performed with a more coordinated manner, it will be more beneficial to the premature neonate infant [14]. Tactile stimulation refers to a sensory stimulation related to the act of touching and is also the process of using touch through a variety of massage strokes [15, 16]. Likewise, auditory stimulation is also recommended as a tool for relieving pain and stress, as it provides an enjoyable strategy for improving compassion and empathy without interfering with routine work in the neonatal intensive care unit [17]. Auditory stimulation is any intentionally made sound described in terms of harmonies, pleasing, tempo, dynamics, rhythm, and volume that activates the auditory system. Mother’s voice is a type of auditory stimulation intervention that is described as playing the previously captured voice of newborn’s mother while performing procedures for preterm neonates [18].

An estimated 15 million children worldwide, or more than 1 in 10 births, are born before their due date each year [1]. The existing worldwide predictable preterm birth incidence rate is approximately 10.6%, and in all countries, it is constantly increasing [19]. In the Middle East, Egypt has reported an incidence of 12% of premature infants [20]. The number of admitted neonates at the NICU of Beni-Suef University Hospital was 1023 in 2023, with an average of 85.25 per month, and 28 of them were preterm neonates, accounting for 32.84% of all neonates as reported by Medical Records of Beni-Suef University Hospital [21]. Compared to others, hospitalized preterm newborns experience invasive, unpleasant procedures 93 times more frequently [12, 22]. In Egypt, limited researches were performed that concentrated on applying sensory stimulation interventions for controlling pain in preterm infants undergoing painful invasive procedures and this may be due to the difficulty and complexity of studying such topics, which require precision in application and experimentation, as well as exactness in observing and measuring their effect. Therefore, this present study was undertaken to evaluate probable positive effects of applying tactile and auditory stimulations on neurobehavioral outcomes and physiological stability of preterm infants. Hoping, the outcomes will set standards. Given this, the study aimed to determine the effect of tactile versus auditory stimulation on reducing pain associated with invasive procedures among preterm neonates at NICUs, with three hypotheses constituting the core of this research. (1) Preterm neonate who receive tactile stimulation will exhibit lower pain scores than those who receive routine care of the unit only; (2) Preterm neonates who receive auditory stimulation will exhibit lower pain scores than those who receive routine care of the unit only; and (3) Preterm neonates who receive tactile stimulation will exhibit lower pain scores than those who receive auditory stimulation.

### Study design and setting

A comparative (three-group) quasi-experimental research design was applied. Per the Transparent Reporting of Evaluations with Non-randomized Designs (TREND) guidelines, a non-randomized trial occurred throughout this study.

This research was executed at the NICU within Beni-Suef University Hospital, which offers round-the-clock care and admits children needing level II and III medical attention. This unit retains a nurse-to-patient ratio of 1:1, offering customized and focused care for preterm, full-term, and post-term neonates with various medical conditions. As to the hospital’s 2023 statistical report, the number of babies admitted to the NICU was 1023 cases. The study went out from the beginning of March to the end of August 2024. The researcher conducted data collection in the NICU from 9:00 a.m. to 3:00 p.m., two days a week (Saturdays and Wednesdays) over six months. This schedule was based on the hospital’s preset timetable, which designated these two days as emergency periods due to the high admission rate of preterm neonates to the NICU. Moreover, most invasive procedures are performed during the morning shift according to hospital policy.

### Participant and sampling approach

**Fig. 1:**
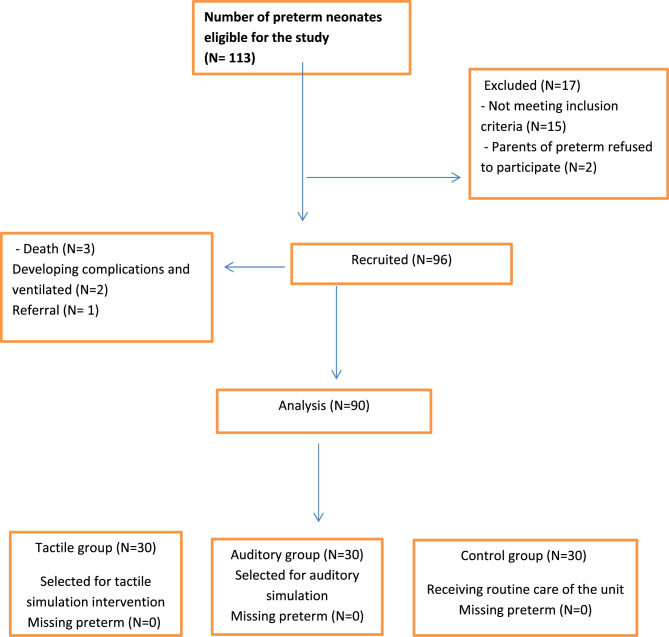
Sample of the study

This study followed a purposive sample of ninety (90) preterm neonates chosen from the above mentioned setting that was enrolled in this study. The application G*Power Windows 3.1.9.7 was applied to determine the subjects using the following settings: power (1-β err prob) = 0.95, α err prob = 0.05, and effect size = 0.5. It exposed a minimal sample size of 90 preterm; therefore, 90 preterm neonates were recruited in this study and selected based on parental consent and specific inclusion criteria. Eligible neonates were at least 2 days old to ensure the resolution of any immediate effects from birth trauma and the influence of maternal analgesia or anesthesia during labor. Additional criteria included their gestational age from 28 to 37 weeks and being free from congenital malformations, not requiring mechanical ventilation, having a birth weight of at least 1000 g, and not receiving any sedatives. Moreover, preterm neonates undergoing only minor invasive procedures were included; look at Fig. 1. Preterm neonates were distributed based on the identification numbers of their cases and divided into three equal groups of 30 preterm neonates. Selection of neonates for the control group was completed first, and then preterm neonates for the other two groups were chosen alternately.

### Instruments

Two instruments were used to gather data for this study.

#### Instrument (I): characteristics and clinical history of preterm neonates

The authors created this instrument according to pertinent research to collect the demographic and clinical history of preterm neonates. This instrument incorporated two parts as follows:

- Part I: Characteristics of preterm neonate such as: postnatal age, gender, birth weight.
- Part II: Clinical history of preterm neonates, such as type of delivery, gestational age, current diagnosis, type of invasive procedure, number of attempts to achieve the procedure, length of hospital stay, and status of preterm neonates on discharge.

#### Instrument (2): Neonatal Infant Pain Scale (NIPS)

This scale was developed by [23]. It is an observational tool that uses six physiological and behavioral indicators for measuring procedural pain in neonates (both preterm and full-term). It consists of facial expression, crying, breathing patterns, arm movements, leg movements, and the state of arousal parameters. It was updated by The Children’s Hospital Medical Center at the University of California, San Francisco (UCSF) in 2005 [24]. Also to include heart rate and oxygen saturation parameters to become an eight-item scale. The researcher adopted this tool to assess preterm neonatal pain by evaluating and reporting the behavioral and physiological indicators four times before, during, immediately after, and after five minutes of each invasive procedure. It is a two-point Likert scale (0–1) except heart rate and crying are a three-point Likert scale (0, 1, and 2). Facial expression was scored (0) if relaxed and scored (1) if grimacing. Breathing patterns were scored (0) if relaxed and (1) if there was a change in breathing. Arm movements were scored (0) if relaxed or restrained and (1) if flexed or extended. Leg movements were scored (0) if relaxed or restrained and (1) if flexed or extended. State of arousal was scored (0) if sleep or awake and (1) if fussy. Cry was scored (0) if no cry, (1) if whimper, and (2) if vigorous crying. Oxygen saturation was scored (0) if no additional oxygen was needed to maintain O2 concentration and (1) if additional oxygen was required to maintain O2 concentration. Heart rate was scored (0) if the increase was within 10% of baseline, (1) if the increase was 11–20% of baseline, and (2) if it was > 20% of baseline. The total pain score ranges from zero to 10; a score of zero represents no pain, a 1–3 score represents mild pain, a 4–6 score represents moderate level of pain, and a 7–10 score represents severe level of pain. The original research revealed strong internal consistency and verified the scale’s multidimensional structure; Cronbach’s alphas were.95 before the procedures, 0.87 during them, and.88 following them [23]. While the α values for NIPS in this study before, during, and after the procedure were.96, 0.88, and.89, respectively.

### Ethical aspects

Official acceptance to carry out the proposed study was secured from the Scientific Research Ethics Committee approval ID: 351,072,023. Participation was entirely voluntary, and each parent of a preterm neonate received comprehensive information about the study before signing the informed consent. Ethical considerations involved clarifying the study’s purpose and nature, highlighting the option to withdraw at any point, and ensuring the confidentiality of all information.

### Data gathering procedure

The researchers obtained permission to collect data by visiting the NICU and identifying the most suitable times to attend during invasive procedures. Data collection was conducted at the NICU from 9:00 a.m. to 3:00 p.m., twice a week (Saturday and Wednesday), over six months. Before initiating data collection, the researchers met with each preterm neonate’s parent and assigned nurse to explain the study’s objectives, seek their cooperation, and reassure them of the confidentiality and scientific use of the information gathered. Based on baseline data from the assessment phase and relevant literature, a study plan was designed. Preterm neonates were distributed into three equal groups of 30 neonates each: a tactile stimulation group, an auditory stimulation group, and a control group. The tactile stimulation group received tactile stimulation alongside routine unit care, while the auditory stimulation group received auditory stimulation in addition to routine care; the control group received routine care only. The selection of neonates for the control group was completed first, and then preterm neonates for the other two groups were chosen alternately. To assess oxygen saturation and heart rate, an appropriate pulse oximeter was selected for use with each neonate in the three groups. The researcher responsible for performing the stimulation is a dedicated practitioner who holds a PhD degree with more than ten years of experience in NICU work in various academic and healthcare settings, such as teaching nursing and caring for neonates. Also the researcher trained a qualified nurse who holds a master’s degree in pediatric nursing on the proper administration of the NIPS to only aid in data collection for the tactile stimulation group. All pain assessments were completed by the same researcher and nurse to ensure consistency. Additionally, the researcher completed instrument one to record each neonate’s personal characteristics and medical history, which took approximately 2–3 min per neonate. For NIPS items (Instrument Two), preterm neonates were observed at various intervals before, during, immediately after, and after five minutes of an invasive procedure during the morning shift in the NICU. An initial pain assessment was conducted before exposure to the invasive procedure to establish baseline data and this was done to ensure that the pain degree during the invasive procedure was clearly indicated.

### For the tactile stimulation group

Tactile stimulation began five minutes prior to the invasive procedure and continued throughout the procedure, immediately after, and up to five minutes post-procedure. Preterm neonates were positioned on their sides, and slow, circular light pressure was applied to the back using warm fingers or the palm in a downward motion, starting from the neck, moving across the shoulders, and then down from the shoulder and upper back to the waist. While the researcher administered the tactile stimulation, a trained nurse, whose primary role was limited to assisting with the NIPS data collection, recorded the required assessment data. And based on the researcher’s observation during the intervention, a double check with the nurse was done, and the recorded data were revised for accuracy. Pain assessments were conducted at intervals: prior to, throughout the procedure, immediately afterward, and five minutes following the invasive procedure.

### For the auditory stimulation group

Auditory stimulation began five minutes prior to the invasive procedure and continued throughout, immediately afterward, and for an additional five minutes post-procedure. The preterm neonate was exposed to a recorded mother’s voice with a steady rhythm and volume, played on a mobile device. Using the same mobile device, the sound volume was adjusted at a moderate level and the device was placed 50 cm away from each preterm neonate. To control environmental noise, the researcher selected quiet zones with soundproofing measures, such as curtains, and conducted auditory stimulation during low-activity periods to minimize external disturbances. Additionally, the incubators in this NICU provide some sound insulation against external noise. Staff was informed about the importance of maintaining a quiet environment, and machine alarms were lowered to the minimum necessary volume. The researcher personally recorded the necessary assessment data. Pain assessments were conducted at specified intervals: prior to, throughout the procedure, immediately after, and five minutes following the procedure.

For both the tactile and auditory groups, the entire process of completing both the intervention and the pain assessment scale took approximately 20 to 30 min per neonate. The intervention applied 5 min prior to the procedure continued throughout the procedure that usually took 3–5 min, immediately after the procedure that took 2–3 min, and after 5 min of the procedure, and recording the obtained data took about 5–10 min.

### For control group

The control group received only the routine care provided in the unit. The researcher ensured that no external sounds or touch were introduced during the procedure for the control group to maintain the integrity of the routine care condition, unnecessary handling beyond routine care was avoided, and no recorded voices were played near the infants. Pain assessments were conducted prior to, during the invasive procedure, immediately afterward, and five minutes post-procedure. The time required to complete the pain assessment scale for this group was approximately 10 to 20 min per neonate.

### Data analysis

Statistical analyses were conducted using SPSS for Windows (version 28). Continuous variables followed a normal distribution and were reported as mean ± standard deviation (SD). Categorical variables were presented as frequencies and percentages. Comparisons among groups were carried out using ANOVA, Pearson’s chi-square, and Kruskal-Wallis tests. All three groups were homogeneously distributed. Correlations between continuous variables were evaluated using the correlation coefficient test. Cronbach’s alpha was calculated to assess the internal consistency of the study tools. A p-value of less than 0.05 was considered statistically significant, whereas a p-value of more than 0.05 indicates the result is not statistically significant. Based on Cohen’s criteria, an effect size of less than 0.01 indicates a small effect, a value between 0.06 and slightly less than 0.14 represents a medium effect, and an effect size of 0.14 or more denotes a large effect.

## Results

Table 1 shows that there is no statistically significant difference between preterm neonate characteristics in the control group, tactile stimulation group, and auditory stimulation group regarding their age, gender, and birth weight (*p* = 0.0213, 0.842, and 0.362, respectively). The median postnatal ages of preterm neonates were 5.0, 4.5, and 4.0 days for the control group, tactile stimulation group, and auditory stimulation group, respectively. Regarding preterm neonates’ gender, half of them were female in the control group, and more than half (56.7%) of them were female in both the tactile stimulation group and the auditory stimulation group. The median birth weight of preterm neonates was 1600 g for the control group, tactile stimulation group, and auditory stimulation group.

**Table 1:** Distribution of the descriptive characteristics of preterm neonates

Group	Control Group (*n*=30)		Tactile stimulation Group (*n*=30)		Auditory stimulation group (*n*=30)		Z*	*P*-value
	Median		Median		Median			
Postnatal age	5.0		4.5		4.0		1.576	0.213
	N	%	N	%	N	%		
Gender								
Male	15	50.0	13	143.3	13	43.3	1.73	0.842
Female	15	50.0	17	56.7	17	56.7		
Birth weight/gm	Median 1600		Median 1600		Median 1600		1.027	0.362

* Z; Kruskal Wallis Test

Table 2 demonstrates that the majority (80.0%) and less than three quarters (73.3%) of the preterm neonates were delivered with cesarean delivery in the tactile stimulation group, auditory stimulation group, and control groups, respectively. The median gestational ages were 32.0, 32.0, and 31.0 weeks for the control group, tactile stimulation group, and auditory stimulation group, respectively. Regarding the current diagnosis, more than half (53.3%, 43.3%, and 40.0%) of preterm neonates had RDS in the control group, tactile stimulation group, and auditory stimulation group, respectively. The most common invasive procedure for preterm neonates was blood sampling, which was done to half (50.0%) of them in the control group, more than half (53.3%) of them in the tactile stimulation group, and more than one third (36.7%) in the auditory stimulation group. The mean number of attempts to achieve the procedure was 2.67 ± 1.093, 3.03 ± 1.098, and 2.33 ± 1.348 for the control group, tactile stimulation group, and auditory stimulation group, respectively. Moreover, the mean length of hospital stay was 5.13 ± 3.702, 4.70 ± 2.756, and 3.73 ± 1.982 for the control group, tactile stimulation group, and auditory stimulation group, respectively. The majority (83.3%) of preterm neonates were improved at discharge in the control group; most (90.0%) of preterm neonates were improved at discharge in each tactile stimulation group and auditory stimulation group.

**Table 2:** Distribution of the clinical history of preterm neonates

**Group**	**Control Group (** ***n*** **=30)**		**Tactile stimulation Group (** ***n*** **=30)**		**Auditory stimulation (** *n* **=30)**	
	**N**	**%**	**N**	**%**	**N**	**%**
Type of delivery						
Normal	8	26.7	6	20.0	8	26.7
Caesarian	22	73.3	24	80.0	22	73.3
Gestational age	**Min **	**Max **	**Min**	**Max **	**Min**	**Max**
Median	28	36	28	36	28	36
	32.0		32.0		31.0	
Current diagnosis						
Hypothermia	-	-	1	3.3	2	6.7
ABO incompatibility	1	3.3	-	-	-	-
RDs	16	53.3	13	43.3	12	40.0
Jaundice	8	26.7	10	33.3	8	26.7
IDM	1	3.3	2	6.7	2	6.7
Necrotizing enterocolitis	1	3.3	-	-	-	-
Neonatal sepsis	-	-	3	10.0	6	20.0
Neonatal seizure	3	10.0	1	3.3	-	-
Type of invasive procedure						
Blood sampling	15	**50.0**	16	**53.3**	11	36.7
Cannulation	8	26.7	8	26.7	8	26.7
Orogastric tube insertion	3	10.0	2	6.7	2	6.7
Suctioning	3	10.0	4	13.3	6	20.0
Random blood glucose monitoring	1	3.3			3	10.0
Number of attempts to achieve the procedure	**Mean score**	**SD**	**Mean score**	**SD**	**Mean score**	**SD**
	2.67	1.093	3.03	1.098	2.33	1.348
Length of hospital stay	5.13	3.702	4.70	2.756	3.73	1.982
Status on discharge						
Death	3	10.0	2	6.7	1	3.3
Improvement	25	83.3	27	90.0	27	90.0
Referral	2	6.7	1	3.3	2	6.7

Table 3 illustrates that the lower mean score of preterm neonates during the procedure was at the tactile stimulation group (0.30 ± 0.466, 0.37 ± 0.490, 0.53 ± 0.507, and 0.43 ± 0.504) for facial expression, breathing pattern, arm, and leg movements, respectively. Moreover, a statistically significant difference between the mean score of these previously mentioned parameters in three groups during, immediately after, and after 5 min of the procedure (*p* < 0.05), while no statistically significant difference between them in the three groups before the procedure. The post-hoc test (b < c < a) represents that the tactile stimulation group experienced the most significant decrease in pain-related responses, followed by the auditory stimulation group, while the control group showed the uppermost distress levels. These results support the hypothesis that both tactile and auditory stimulation can effectively reduce preterm pain responses, with tactile stimulation having the greatest effect.

**Table 3:** Comparison of mean scores of facial expression, breathing patterns, and arm and leg movements throughout the procedure

Parameters	Control Group (*n*=30)		Tactile stimulation Group (*n*=30)		Auditory stimulation (*n*=30)		Kruskal Wallis Test		Post-hop Test
	Mean score	SD	Mean score	SD	Mean score	SD	Z	*P*-value	
Facial expression									
Before	0.03	0.783	0.07	0.254	0.10	0.305	1.060	0.589	a<b<c
During	0.90	0.305	0.30	0.466	0.63	0.490	22.562	0.000**	b<c<a
Immediately after	0.77	0.430	0.07	0.254	0.73	0.450	37.080	0.000**	b<c<a
After 5 min.	0.47	0.507	0.00	0.000	0.20	0.407	18.817	0.000**	b<c<a
Breathing pattern									
Before	0.03	0.183	0.03	0.183	0.07	0.254	0.517	0.772	a<b<c
During	0.80	0.407	0.37	0.490	067	0.479	12.298	0.002**	b<c<a
Immediately after	0.73	0.450	0.23	0.430	0.40	0.498	15.505	0.000*	b<c<a
After 5 min.	0.67	0.479	0.00	0.00	0.10	0.305	40.313	0.000**	b<c<a
Arm movements									
Before	0.07	0.254	0.03	0.183	0.00	0.0.00	2.046	0.360	a<b<c
During	0.93	0.254	0.53	0.507	0.67	0.479	11.981	0.003**	b<c<a
Immediately after	0.73	0.450	0.33	0.479	0.43	0.504	10.284	0.006**	b<c<a
After 5 min.	0.50	0.509	0.13	0.063	0.17	0.069	12.473	0.002**	b<c<a
Leg movements									
Before	0.03	0.183	0.00	0.000	0.13	0.346	5.445	0.066	b<a<c
During	0.87	0.346	0.43	0.504	0.67	0.479	12.36	0.002**	b<c<a
Immediately after	0.80	0.407	0.27	0.450	0.50	0.509	16.999	0.000**	b<c<a
After 5 min.	0.53	0.507	0.00	0.000	0.13	0.346	26.446	0.000**	b<c<a

*Z* Kruskal Wallis Test, *S.D* stander deviation

**p-value < 0.05 significant, *p*-value >0.05 not significant

Table 4 clarifies that the lower mean score of preterm neonates during the procedure was at the tactile stimulation group (0.7 ± 0.450, 0.63 ± 0.615, 0.37 ± 0.490, and 0.60 ± 0.498) for state of arousal, cry, oxygen saturations, and heart rate, respectively. The Kruskal-Wallis test findings disclose significant differences in these previously mentioned parameters among the three groups during, immediately after, and five minutes post-stimulation (*p* < 0.05). According to the post-hoc test (b < c < a), the tactile stimulation group experienced the greatest reduction in distress markers, followed by the auditory stimulation group. In contrast, the control group exhibited the highest distress levels. According to these results, neonatal stress responses can be successfully reduced by both tactile and auditory stimulation, with tactile stimulation exhibiting the greatest advantages.

**Table 4:** Comparison of mean scores of state of arousal, cry, oxygen saturations, and heart rate throughout the procedure

Parameters	Control Group (*n*=30)		Tactile stimulation Group (*n*=30)		Auditory stimulation (*n*=30)		Kruskal Wallis Test		Post-hop Test
	Mean score	SD	Mean score	SD	Mean score	SD	Z	*P*-value	
State of arousal									
Before	0.03	0.183	0.00	0.000	0.03	0.183	1.011	0.603	b<a<c
During	0.87	0.346	0.7	0.450	0.43	0.504	22.811	0.000**	b<c<a
Immediately after	0.80	0.407	0.23	0.430	0.60	0.498	19.758	0.000**	b<c<a
After 5 min.	0.53	0.507	0.00	0.000	0.23	0.430	22.293	0.000**	b<c<a
Cry									
Before	0.07	0.54	0.10	0.305	0.10	0.305	0.271	0.873	a<b<c
During	1.73	0.450	0.63	0.615	1.03	0.669	34.874	0.000**	b<c<a
Immediately after	1.33	0.661	0.23	0.430	0.70	0.535	35.736	0.000**	b<c<a
After 5 min.	0.80	0.551	0.07	0.254	0.37	0.490	28.466	0.000**	b<c<a
Oxygen saturations									
Before	0.53	0.507	0.30	0.466	0.30	0.466	4.581	0.101	b<c<a
During	0.77	0.430	0.37	0.490	0.83	0.379	16.739	0.000**	b<c<a
Immediately after	0.60	0.498	0.30	0.466	0.73	0.450	11.784	0.003**	b<c<a
After 5 min.	0.50	0.509	0.33	0.479	0.50	0.509	2.225	0.329	b<c<a
Heart rate									
Before	0.00	0.000	0.07	0.254	0.07	0.254	2.070	0.355	a<b<c
During	1.60	0.621	0.60	0.498	0.63	0.615	28.671	0.000**	b<c<a
Immediately after	1.30	0.596	0.27	0.716	0.63	0.669	32.355	0.000*	b<c<a
After 5 min.	0.83	0.531	0.07	0.254	0.10	0.305	43.462	0.000**	b<c<a

*Z* Kruskal Wallis Test, *S.D* stander deviation

**p-value < 0.05 significant, *p*-value >0.05 not significant

Table 5 illuminates that the total NIPS mean score during the procedure was 8.47 ± 1.196 in the control group, 3.50 ± 1.225 in the tactile stimulation group, and 5.57 ± 1.431 in the auditory stimulation group. The finding represent a significant difference in neonatal infant pain scores amongst the three groups at all-time points (*p* < 0.05). Before stimulation, the effect size was small, representing little difference between groups. However, during, immediately after, and five minutes post-stimulation, the effect sizes were large (Cohen’s d > 0.7), implying a significant effect of tactile and auditory stimulation on pain reduction. The post-hoc test results (b < c < a) confirm that the tactile stimulation group experienced the greatest significant pain reduction, followed by the auditory stimulation group, while the control group showing the highest pain scores.

**Table 5:** Comparison of mean scores of the total neonatal infant pain scale (NIPS) throughout the procedure

	Control Group (*n*=30)		95% confidence interval for mean		Tactile stimulation group (*n*=30)		95% Confidence interval for mean		Auditory stimulation (*n*=30)		95% Confidence interval for mean		One Way ANOVA Test		Post-hop Test	Effect size	
																Cohen's	Level
	Mean score	SD	Lower Bound	Upper Bound	Mean score	SD	Lower Bound	Upper Bound	Mean score	SD	Lower Bound	Upper Bound	t	*P*-value			
Total neonatal infant pain																	
Before	0.80	0.714	.530	1.07	0.60	0.675	.350	0.85	0.80	0.761	.520	1.08	0.777	0.463	b<a<c	0.0175	Small
During	8.47	1.196	8.02	8.91	3.50	1.225	3.04	3.96	5.57	1.431	5.03	6.10	112.564	0.000*	b<c<a	0.721	Large
Immediately after	7.07	1.202	6.62	7.52	1.93	1.363	1.42	2.44	4.73	1.081	4.33	5.14	133.036	0.000*	b<c<a	0.754	Large
After 5 min.	4.83	1.315	4.34	5.32	0.60	0.563	.390	.810	1.80	1.297	1.32	2.28	114.865	0.000*	b<c<a	0.725	Large

One Way ANOVA Test; S.D= stander deviation

*p-value < 0.05 significant, *p*-value >0.05 not significant. Cohen's criteria <0.01 small effect, 0.06-<0.14 medium effect, ≥ 0.014 large effect

**Fig. 2:**
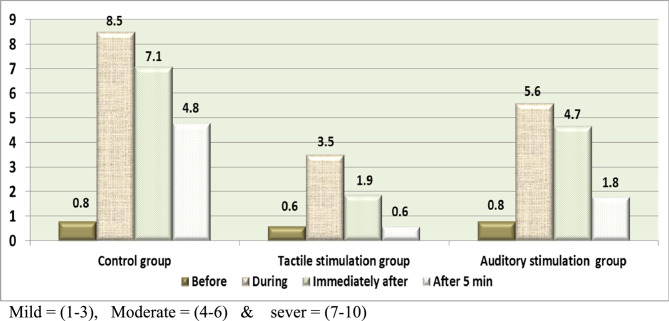
Mean score of the NIPS throughout the procedure

Figure 2 Illustrates that the preterm neonates had a mild level of pain during the procedure at the tactile stimulation group with a mean score of 3.5, and preterm neonates had a moderate pain during the procedure at the auditory stimulation group with a mean score of 5.6, while preterm neonates had a severe level of pain during the painful procedure at the control group with a mean score of 8.5. Preterm neonates in the tactile stimulation group showed lower total NIPS mean scores than preterm neonates in the control group and auditory stimulation group during, immediately after, and after 5 min of the procedure.

Table 6: The Pearson Correlation Coefficient results reveal varying associations between neonatal pain responses and age, gender, and birth weight across the three groups. Age was found to significantly positively correlate with pain levels in the tactile stimulation group both before (*r* = 0.409, *p* = 0.025) and after five minutes (*r* = 0.631, *p* = 0.000). This suggests that older neonates may benefit more from tactile stimulation in terms of pain alleviation. In the control group, birth weight confirmed a significant negative correlation with pain scores during (*r* = −0.459, *p* = 0.011) and immediately after (*r* = −0.475, *p* = 0.008), denoting that lower birth weight neonates may show higher pain responses. Likewise, in the auditory stimulation group, birth weight presented a significant negative correlation with pain scores during (*r* = −0.562, *p* = 0.001), immediately after (*r* = −0.394, *p* = 0.031), and after five minutes (*r* = −0.453, *p* = 0.012). These results demonstrate the potential impact of neonatal characteristics on pain perception and response to different stimulation methods.

**Table 6:** Correlation between the preterm infants' characteristics and their total neonatal infant pain before, during, immediately after, and after 5 minutes of procedure

	Age		Gender		Birth weight	
	r	P	r	P	r	P
Before						
Control group	0.052	0.784	0.095	0.618	-0.104	0.585
Tactile stimulation group	0.409	0.025*	-0.223	0.236	-0.203	0.281
Auditory stimulation group	-0.257	0.170	0.216	0.252	-0.187	0.324
During						
Control group	0.016	0.934	0.227	0.228	-0.459	0.011*
Tactile stimulation group	0.141	0.456	-0.091	0.632	-0.148	0.0.435
Auditory stimulation group	-0.274	0.143	-0.030	0.874	-0.562	0.001*
Immediately after						
Control group	0.095	0.618	0.339	0.067	-0.475	0.008*
Tactile stimulation group	-0.246	0.189	0.258	0.169	0.240	0.201
Auditory stimulation group	-0.107	0.575	0.350	0.058	-0.394	0.031*
After 5 min						
Control group	0.161	0.396	-0.129	0.497	-0.136	0.473
Tactile stimulation group	0.631	0.000*	0.097	0.609	-0.157	0.408
Auditory stimulation group	-0.186	0.324	0.127	0.505	-0.453	0.012*

Pearson Correlation Coefficient, *Statistically significance p≤ 0.05, *p*-value >0.05 not significant.

## Discussion

Effective pain management mitigates immediate discomfort and potential long-term developmental consequences. Non-pharmacological interventions, such as tactile and auditory stimulation, have been explored as potential methods to reduce neonatal pain during invasive procedures. Non-pharmacological pain management is a crucial part of nursing intervention at NICU [25]. So, this study aimed to determine the effect of tactile versus auditory stimulation on reducing pain associated with invasive procedures among preterm neonates at NICUs. The findings of the present study highlighted that no statistically significant difference was noticed among the mean score of both the physiological indicators (e.g., oxygen saturation level, heart rate, breathing pattern, and state of arousal) and behavioral responses including facial expression, crying, and arm, and leg movements in the three study groups before the procedure. This result was in accordance with that obtained from [18, 25–27], which pointed out no statistical difference between the mean score of preterm infants, behavioral responses and physiological parameters to painful procedures in all groups of neonates involved in the study before performing the painful procedure. Also, the ongoing study findings revealed that the lower mean score regarding both the physiological parameters and behavioral responses during the procedure was in the tactile stimulation group. And these results were supported by [28–30], who conducted research on the positive impacts of human touch on preterm babies and found that the lower mean score of preterm neonates during unpleasant procedures was in the tactile stimulation group regarding physiological parameters and neonates’ facial expression, body movements, and crying intensity and duration. Additionally, the outcomes of the present study reported that the preterm neonates who were assigned to the tactile stimulation group had lower facial expression, cry, arm movement, and leg movement mean scores than those assigned to the auditory stimulation group and control group during, immediately after, and 5 min after the procedure. It might be justified by tactile stimulation, which possibly motivates a state of calmness and relaxation in newborns, minimizes energy disbursement, and keeps up regular respiration [31]. This result matched that of [32], who mentioned that tactile stimulation (TS) had an improvement effect on preterm infants, including behavioral status. Regarding breathing patterns, oxygen saturations, and heart rates, the outcome of the ongoing study exhibited that premature newborns fall within the tactile stimulation group had lower mean scores than preterm newborns assigned to the auditory stimulation group and control group during, immediately after, and 5 min after the procedure. This could be due to the physiology that pain usually results in irregular respiration, and tactile stimulation can calm the preterm infants down; therefore, their oxygen saturation levels are increased while the respiratory rate and heart rate are decreased, thereby promoting physiological stability [33]. Furthermore, tactile stimulation can enhance the release of epinephrine, as well as catecholamine, that affect beta-adrenergic receptors of the airways by widening their diameter. Subsequently, this results in improved oxygen saturation and upgraded alveolar ventilation, as explained via [34]. This result was compatible to [35], who mentioned that gentle touch significantly reduced the mean score of physiological indicators during and following the painful procedure that was assessed using NIPS. Regarding the state of arousal, the findings of the present study presented that preterm neonates within the tactile stimulation group had lower mean scores than those within the control group and auditory stimulation group during, immediately after, and after 5 min of the procedure. This may be interpreted by the fact that tactile/touch stimulation, as a type of massage, has been proven to produce relaxation by lowering norepinephrine and cortisol levels while increasing serotonin and endorphin levels [36]. This finding was aligned with [37], who stated that gentle human touch might have a relaxing, soothing impact on premature infants and can also improve their sleep patterns in the intervention group in contrast to the non-intervention group. Moreover, the present study’s findings indicated that a statistically significant difference was observed between the mean score of both the physiological indicators and behavioral responses in the three study groups during, immediately after, and after 5 min of the procedure. This result was aligned with that obtained from [25, 38, 38, 39], and [40], who clarified that premature infants who were subjected to the study intervention scored significantly better than those subjected to the control group regarding physiological parameters and behavioral responses during and after completing the invasive procedure (*P* < 0.05). On the contrary, this previously mentioned result conflicted with research achieved through [41] and [42], who studied non-pharmacological approaches that minimize preterm neonatal pain and stated that the control group and the experimental group did not differ statistically in terms of physiological indicators and behavioral responses prior to, throughout, and following the procedure (*p* > 0.05). In the researcher’s opinion, this difference might be due to the different applied intervention techniques and the difference in study sample (preterm/preterm and full-term infants) included in each study. Also, the outcomes of the current research clarified that the total NIPS mean score throughout performing the procedure was 3.50 ± 1.225 in the tactile stimulation group, 5.57 ± 1.431 in the auditory stimulation group, and 8.47 ± 1.196 in the control group. These results might be interpreted as tactile stimulation, and a mother’s voice may distract preterm infants from the pain that they experience during painful procedures. We suggest ways to integrate tactile and auditory stimulation into routine neonatal care, such as structured gentle touch, skin-to-skin contact, or soothing auditory interventions during painful procedures. This finding was in concurrence with that of [42, 43]. Additionally, the current study clarified that the total NIPS mean score immediately after the procedure was 1.93 ± 1.363 for the tactile stimulation group, 4.73 ± 1.081 for the auditory stimulation group, and 7.07 ± 1.202 in the control group. The previously mentioned outcome was compatible with the outcomes of [12] and that of [44], who reported that the total NIPS mean score assessed immediately after performing the painful procedure in the intervention group was markedly fewer than that of the non-intervention group. The results of the ongoing research clarified that the total NIPS mean score after five minutes of procedure was 0.60 ± 0.563 in the tactile stimulation group, 1.80 ± 1.297 in the auditory stimulation group, and 4.83 ± 1.315 for the control group. These findings might be interpreted as both tactile and auditory stimulation can decrease pain sensation and encourage relaxation through invoking the parasympathetic nervous system [45]. This outcome was aligned with [46], who conveyed that the total NIPS mean score measured five minutes following the performance of the procedure was 1.15 ± 0.84 in the intervention group and 3.00 ± 0.98 in the control group. Also, the outcomes of the present research reported that preterm newborns subjected to the tactile stimulation group had lower total NIPS mean scores than those subjected to the auditory stimulation group and control group during, immediately after, and 5 min after the procedure. This might be due to the mechanism through which the tactile stimulation technique exerts its analgesic effect by means of contact between skin that induces the production of hormones, including oxytocin, that suppress the sympathetic nervous system, leading to a better stability of the hemodynamic status [47]. This previous finding was similar to that of Efendi, who mentioned that maternal therapeutic touch (MTT) markedly decreased scores of pain in preterm newborns who received the study intervention as opposed to those of the comparison group (*p* < 0.05) at 15–19 min. Furthermore, MTT reduced the scores of pain at 25–27 min (*p* < 0.05) [48]. Also, the existing study findings reported that premature newborns within the auditory stimulation group experienced lower total NIPS mean scores than those within the control group throughout, immediately afterward, and after 5 min of the procedure. This might be explained as listening to the maternal sound creates a secure and safe environment for preterm neonates. The development and neurology of fetuses and neonates are significantly influenced by the mother’s voice. Around 26 to 28 weeks of pregnancy, a fetus is able to respond to sound and discriminate between maternal voice and other noises. A maternal voice has an influence on the physiology of behavior and emotion in premature babies [49]. This study finding was on the same line with research applied to low-birth-weight and premature infants who were exposed to voice recordings of their mothers by Efendi and with that of Ding, who mentioned that mother’s voice stimulus, or MVS, showed a significant reduction in the level of pain in premature newborns throughout painful procedures in contrast to routine care [48] and [50]. Also, this finding was in agreement with the findings of Williamson & McGrath, who carried out a systematic review and noticed that many studies had discussed that the autonomic nerves stability improves due to exposure to the mother’s voice, particularly, the respiration and heart rate [51]. Regarding pain severity between the three study groups, the current study indicated that a significant difference was observed among the total NIPS of the three groups throughout, immediately afterward, and also after 5 min of the procedure. Otherwise, no statistically significant difference was observed between the total NIPS of all groups before performing the procedure. These results were on the same line as the result of Fatollahzade, who showed that a significant difference was noted among the mean pain scores in the non-experimental and experimental cases (*p* < 0.002), and the cases with intervention demonstrated a considerably decreased mean score of pain [52]. And with the outcome of recent research accomplished via Aranha, who showed that no statistically significant difference was observed among the total NIPS of different groups before performing any painful procedure [28]. Moreover, the present study results illustrated that preterm neonates had a mild level of pain during the procedure at the tactile stimulation group with a mean score of 3.5, and preterm neonates had pain of a moderate level while performing invasive procedures at the auditory stimulation group with a mean score of 5.6, while preterm neonates had a severe level of pain during invasive procedures at the comparison group with a mean score of 8.5. This previous result was congruent with that of Walas, who reported that the majority of preterm infants who received tactile stimulation as an intervention to relieve pain during an invasive procedure experienced a mild level of pain, while more than two-thirds of the group that received auditory stimulation had a moderate to severe level of pain, and most of the control group suffered from moderate to severe pain [53]. Thus, the current study hypotheses were asserted, as the premature neonates in the tactile and auditory stimulation groups demonstrated lower pain scores than premature neonates in the control group. The findings of the ongoing research indicated that a statistically significant positive correlation was found between preterm neonates’ age and their NIPS before and after 5 min of procedure in the tactile stimulation group. These results demonstrate the potential impact of neonatal characteristics on pain perception and response to different stimulation methods. This correlation was thought to be due to several factors; first, preterm infants have an immature central nervous system (CNS), which may make them more sensitive to pain and less able to regulate their responses to painful stimuli. Other factors, such as individual differences in pain sensitivity and the type and duration of the painful stimulus, may also affect NIPS scores and pain perception in preterm infants [54]. This study’s findings were consistent with the results of prior research conducted by Menin & Dondi, who mentioned that a positive correlation is present between preterm infants’ age and their NIPS scores, meaning that as the age of the infant decreases, the NIPS scores tend to be higher, indicating higher levels of pain [55].

Finally, these findings suggest that a multimodal approach incorporating various sensory interventions is possibly the most efficient strategy to manage pain in preterm neonates. Therefore, the nurse should be aware of these non-pharmacological management techniques for pain in this vulnerable group. We should conduct further research to develop specialized programs that aim to reduce pain using non-pharmacological methods. These programs will enable an appropriate assessment and management of preterm pain and help to improve their quality of life. Future research should explore the feasibility of integrating tactile and auditory stimulation into standard NICU pain management protocols, comparing their effectiveness to pharmacological interventions. Studies should also assess the impact of these sensory stimulations on neonatal outcomes, including weight gain, feeding tolerance, length of hospital stay, and neurodevelopmental progress. Furthermore, to enhance the generalizability of the findings to other hospital settings, the study must be expanded to multiple hospitals in different geographic regions, including a larger and more diverse sample of preterm infants to provide more representative insights, and conducting research across Level I, II, and III NICUs would help determine whether the findings are consistent across hospitals with varying levels of neonatal care. Lastly, replicating the study in various hospital settings using similar methodologies would validate the results and strengthen their applicability.

### Strengths and limitations of the study

Strengths of this research can comprise the study design; the study examined three different equal groups that enable comparison between positive impacts of distinct sensory stimulations and recommend the best of them for practical use. Also, this study is greatly valuable as it properly quantifies and generalizes perception of pain throughout various invasive painful procedures, therefore displaying a pre-intervention base for anticipated medical procedures. This research also has a significant clinical value in optimizing the clinical pathways of the existing management of preterm newborns’ pain.

However, this study reflects the preterm neonatal pain experience to the greatest extent; it might be noticed that this research has some restrictions and limitations. The main drawback of this study is its lack of randomization, which may create selection bias and influence the generalizability of the results. Confounding effects could arise if certain participant characteristics affected the results without random assignment. To allay this worry, we have meticulously recorded baseline data such as gestational age, clinical condition, and severity of illness and adjusted for important variables in the analysis. Future research should consider using randomized designs to improve internal validity and lessen potential biases. The research was implemented at only one unit within a single hospital setting because this is the only hospital that provided us with formal permission to perform the study, which may limit our capability to generalize findings to other settings, so replicating the study in other geographical settings is essential. In addition to Neonatal Infant Pain Scale (NIPS), several other pain scoring systems are used for assessing pain in preterm neonates. Neonatal Infant Pain Scale (NIPS) was chosen because it is a reliable and widely used tool for assessing pain in neonates, particularly in clinical settings such as the NICU. NIPS provide a practical, non-invasive, and objective method to assess neonatal pain responses during and after invasive procedures. Additionally, its ease of use and applicability in both preterm and full-term infants make it an ideal choice for studies examining the effectiveness of pain management interventions, such as tactile and auditory stimulation. Moreover, the study excluded seriously ill and neurologically impaired preterm neonates, which can restrict the generalizability of the study results to the excluded population groups. Despite the researcher’s best efforts to control the environmental noises, the sounds of the hospital environments can be considered another limitation.

## Conclusion

As regards the current study, both tactile and auditory stimulation can be considered an inexpensive, accessible, and uncomplicated procedure to improve pain in preterm neonates. It was demonstrated that both tactile and auditory stimulation, including maternal recorded voice, could markedly decrease the scores of pain in preterm neonates undergoing painful invasive procedures and are effective in improving neurobehavioral outcomes and physiological stability of preterm.

### Implications to practice

The key components of nursing practice are highlighted in this research. The care and development of preterm newborns in NICUs could be greatly improved by tactile and auditory stimulation. In preterm neonates who are extremely sensitive to environmental stresses, these interventions assist in stabilizing vital indicators including heart rate and oxygen levels by offering gentle touch, skin-to-skin contact, and calming noises or voices. Furthermore, early sensory experiences are critical for brain development, especially for premature babies who miss critical phases of development while still in the womb. By offering controlled sensory input, encouraging longer stretches of sound sleep, and lowering stress, tactile and aural stimulation aids neurodevelopment and improves long-term results. Crucially, bonding and attachment are promoted when parents participate in various types of stimulation. Also, there are still gaps in how neonatal nurses in the NICU care for preterm neonates; thus, they need efficient methods to train neonatal nurses on the application of non-pharmacological measures such as tactile and auditory stimulation to relieve pain during invasive procedures. We suggest methods to add tactile and auditory stimulation into routine neonatal care, such as structured gentle touch, skin-to-skin contact, or soothing auditory interventions during painful procedures, and latent benefits of incorporating these non-pharmacological pain management strategies into existing care frameworks.

## Data Availability

The datasets used or analysed during the study available from the corresponding author on reasonable request.
